# Supplementary figures and images for: Myosin A and F-Actin play a critical role in mitochondrial dynamics and inheritance in Toxoplasma gondii
Source: PLoS Pathog. 2024 Oct 7;20(10):e1012127. doi: 10.1371/journal.ppat.1012127 (PMC11486366; doi:10.1371/journal.ppat.1012127)

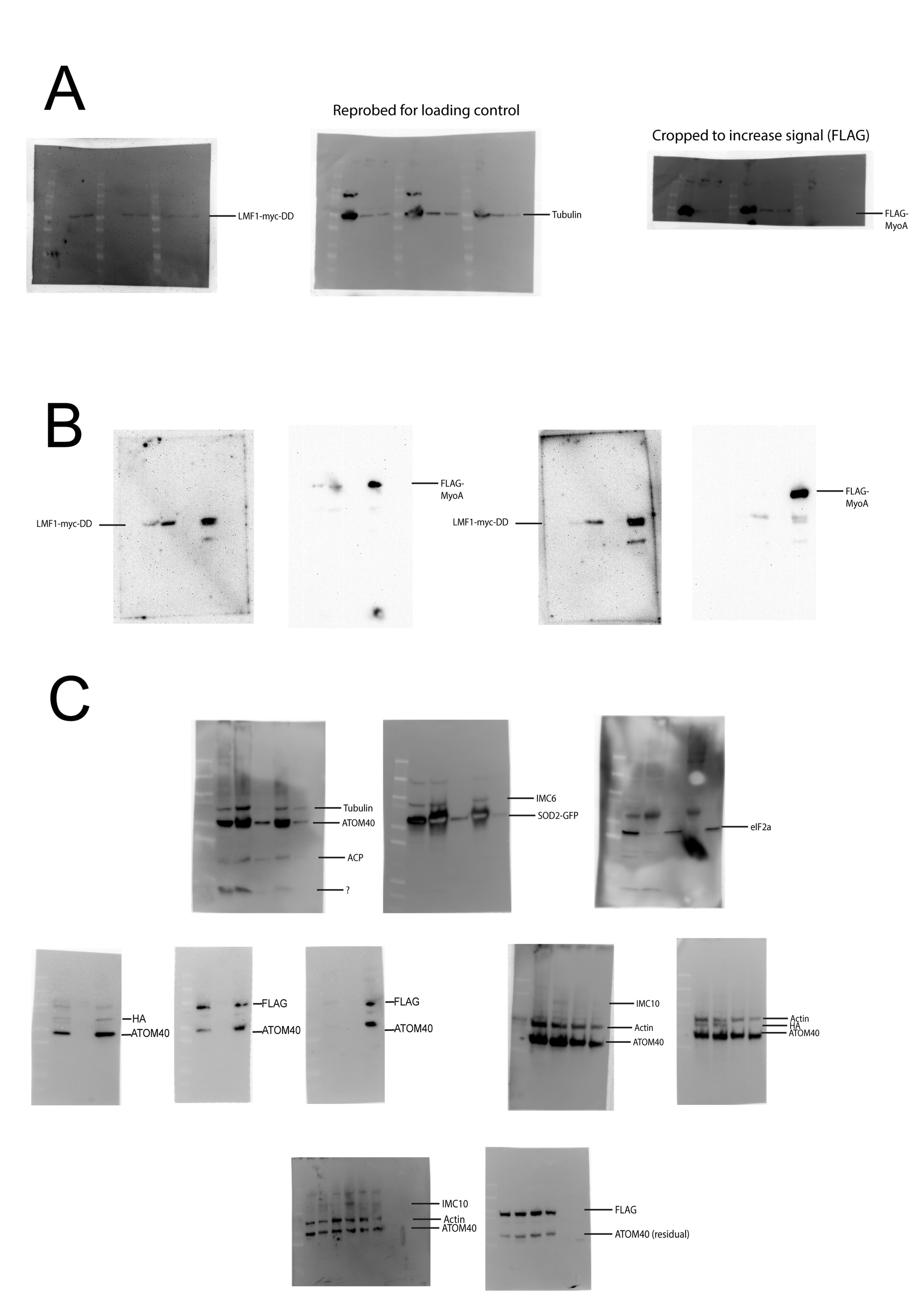

Supplement: S1 Fig — Western blots of Figs 6A, 6B, and 10B. (TIF) [file ppat.1012127.s008.tif]

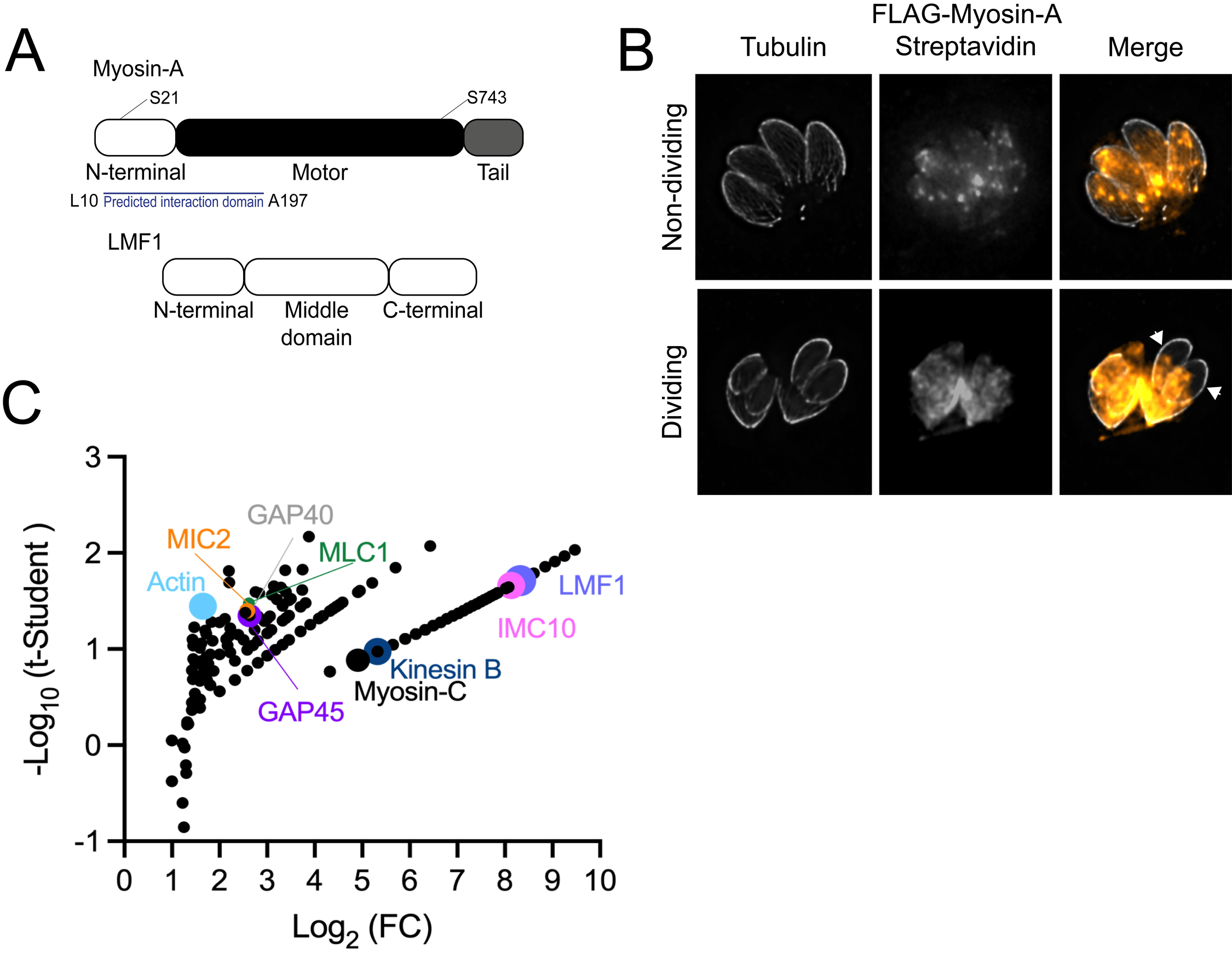

Supplement: S2 Fig — A. Schematic of the three main domains in Myosin-A and LMF1, not to scale. The relative positions of the two phosphorylation sites (S21 and S743) discussed in this work are marked. The region in MyoA found in the yeast two hybrids to interact with LMF1 is highlighted. B. Immunofluorescence assays (IFA) of non-dividing and dividing intracellular FLAG-Myosin-A parasites stained for tubulin (gray) and streptavidin-594 (orange). Arrows point at biotinylated proteins in the emerging daughter cells. C. Mass spectrometry analysis of FLAG-Myosin-A biotinylated proteins isolated using streptavidin beads. Highlighted are proteins known to interact with Myosin-A and proteins involved in mitochondrial dynamics, such as LMF1 (purple) and IMC10 (pink). (TIF) [file ppat.1012127.s009.tif]

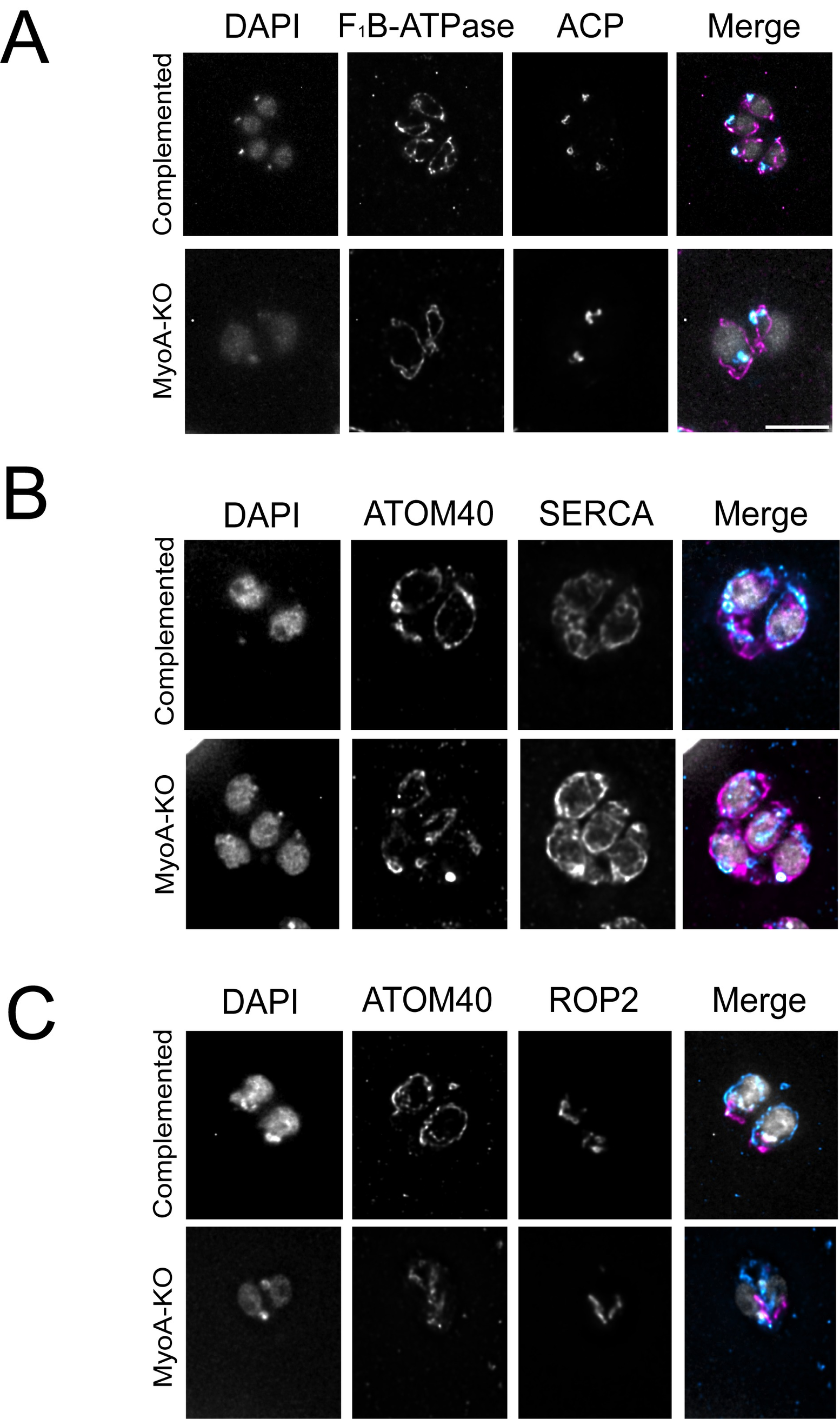

Supplement: S3 Fig — Intracellular parasites of the complement and myosin-A KO strains were grown in fibroblasts for 16 hours, then stained with anti-ACP to detect the apicoplast (A), anti-SERCA to detect the ER (B), or with anti-ROP2 to detect the rhoptriesROP2 (rhoptries). Either F1B-ATPase (A) or ATOM40 (B and C) were used as a mitochondrial marker. Scale bar: 5 μm. (TIF) [file ppat.1012127.s010.tif]

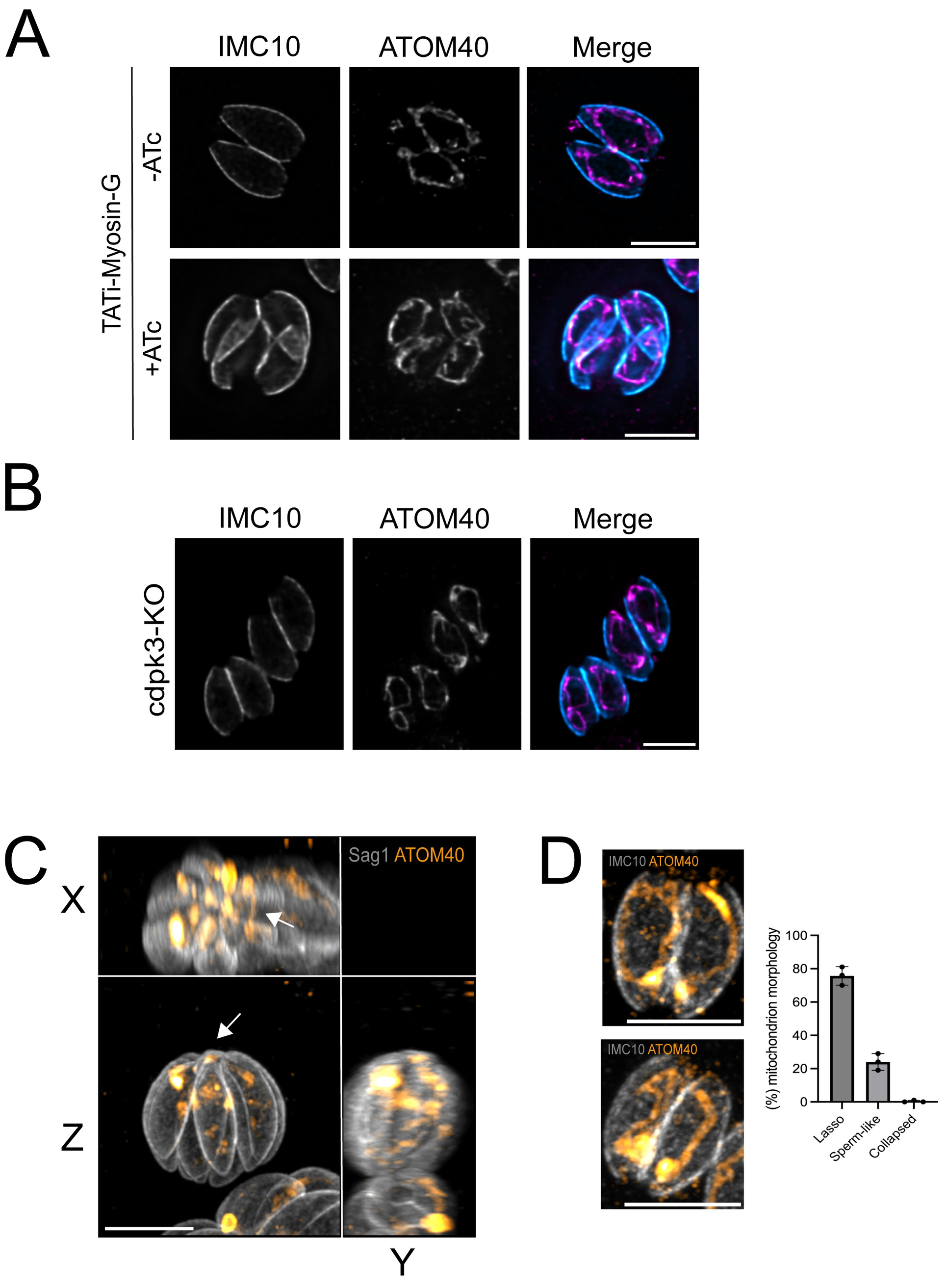

Supplement: S4 Fig — Intracellular parasites of the TATi-Myosin-G (A) and cdpk3-KO (B) were grown in fibroblasts for 16 hours, then stained for IMC10 (cyan) and F1B-ATPase, a mitochondrial marker (magenta). To induce the knockdown of Myosin-G, parasites were kept in the presence of anhydrotetracycline (ATc, 0.5 μg/ml) for 24h. C. Representative IFA showing an xyz view of S21A parasites, confirming an interconnected mitochondrion phenotype. Parasites were stained for SAG1 (gray) and ATOM40 (mitochondrion). D. Representative IFA of S20-21 DD phosphomimetic mutant. Parasites were stained for IMC10 (gray) and ATOM40 (orange). To the right, quantification of the mitochondrion morphology in this cell line. Bar graphs represent mean ±sd of the percentage of parasites showing each morphology. Experiments were performed in biological triplicates and a total of 50 parasites were counted per replicate. Scale bar: 5 μm. (TIF) [file ppat.1012127.s011.tif]

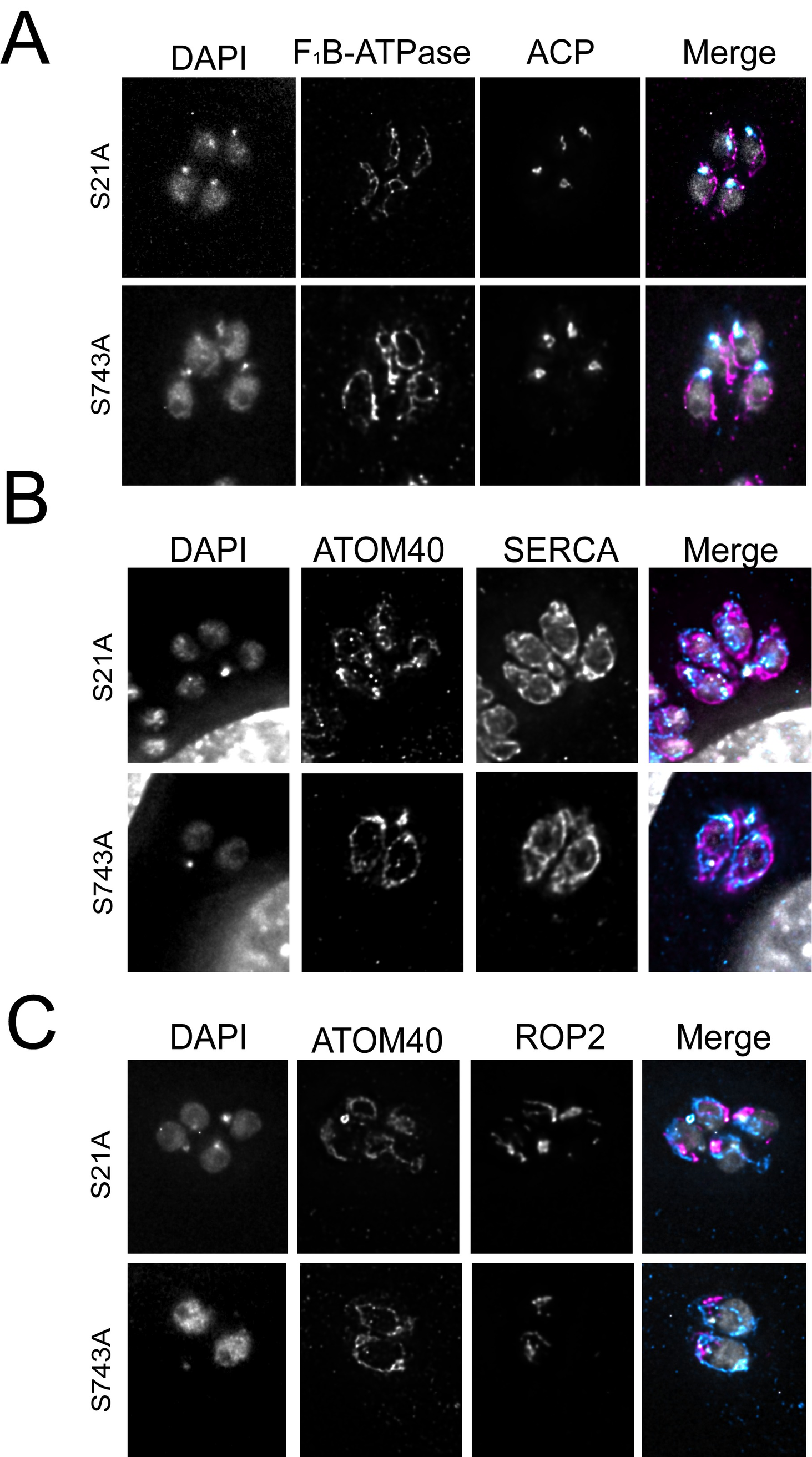

Supplement: S5 Fig — Intracellular parasites expressing FLAG-Myosin-A S21A and FLAG-Myosin-A S743A were grown in fibroblasts for 16 hours.Cultures were then stained with anti-ACP to detect the apicoplast (A), anti-SERCA to detect the ER (B), or with anti-ROP2 to detect the rhoptriesROP2 (rhoptries). Either F1B-ATPase (A) or ATOM40 (B and C) were used as a mitochondrial marker. Scale bar: 5 μm. (TIF) [file ppat.1012127.s012.tif]

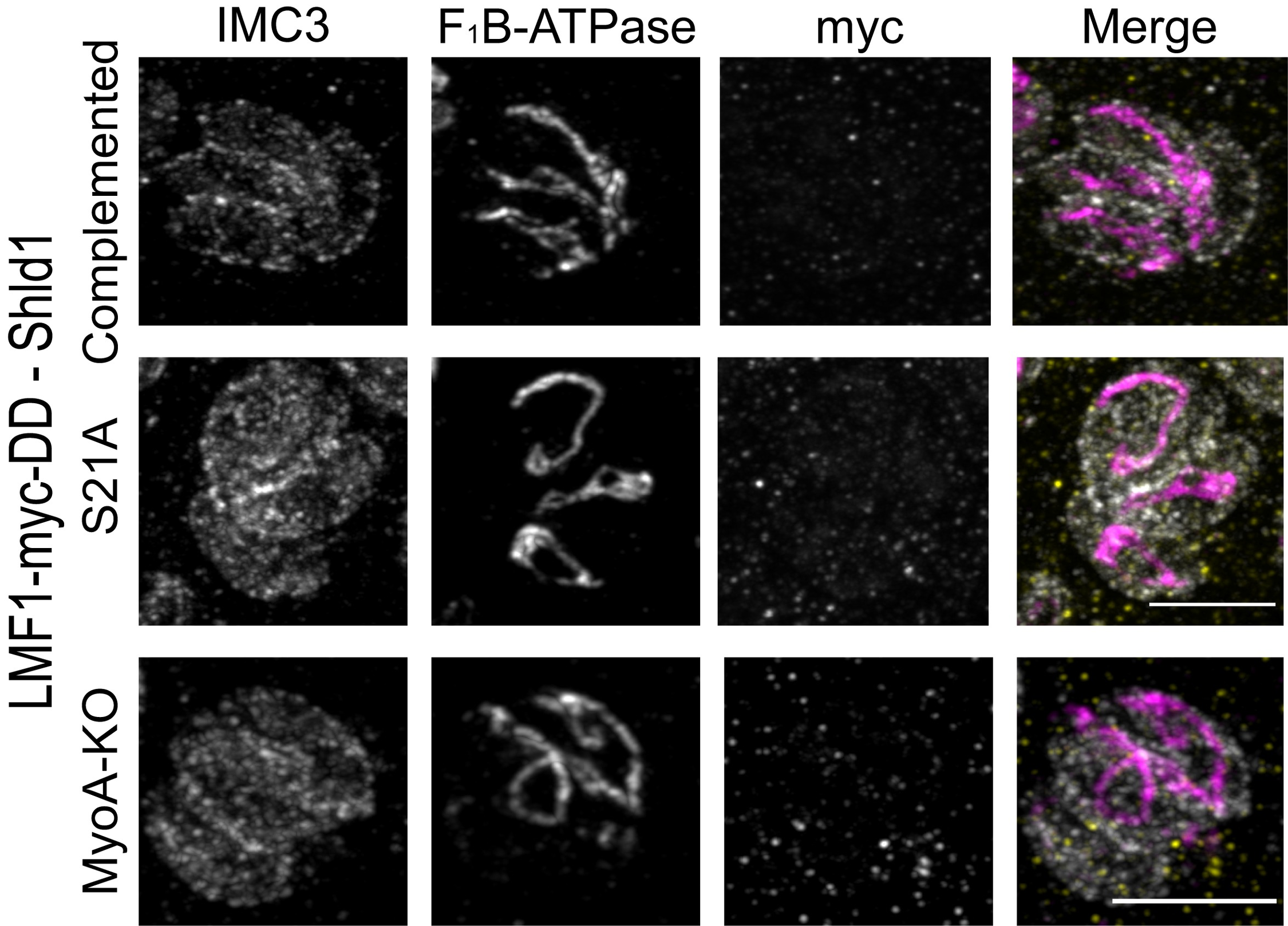

Supplement: S6 Fig — Representative IFA of MyoA complemented, MyoA S21A or MyoA KO parasites expressing LMF1-myc-DD showing expression LMF1(Myc) in the absence of Shld1. Parasites were stained for IMC3 (gray), F1B-ATPase (magenta), and myc (yellow). Scale bar: 5μm. (TIF) [file ppat.1012127.s013.tif]

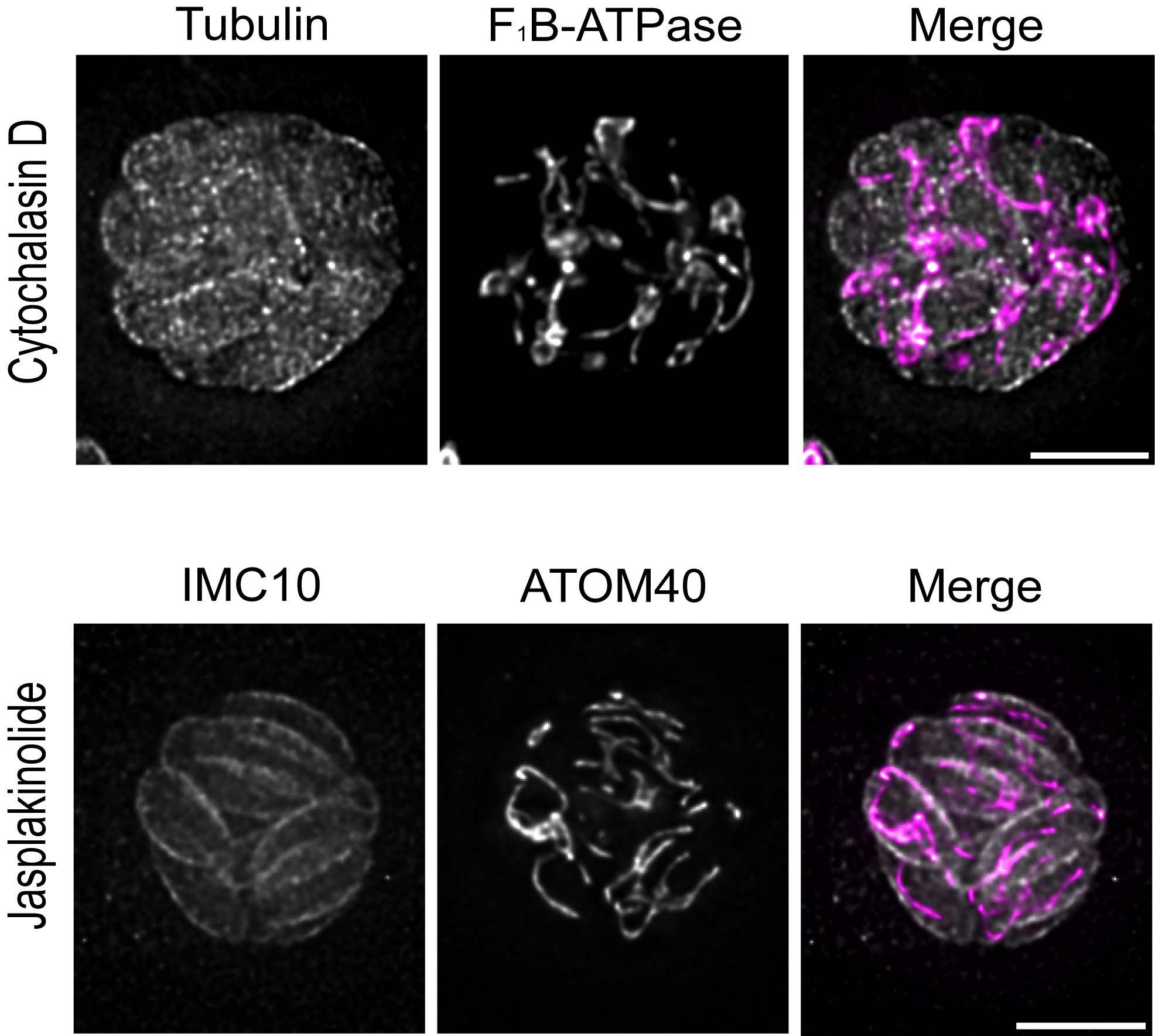

Supplement: S7 Fig — IFA of large parasite vacuoles grown in the presence of 1 mM of Cytochalasin D (A) or 1 mM Jasplakinolide (B). Parasites were stained for Tubulin (gray) and F1B-ATPase (magenta) or IMC10 (gray) and ATOM40 (magenta). Scale bar: 5μm. (TIF) [file ppat.1012127.s014.tif]

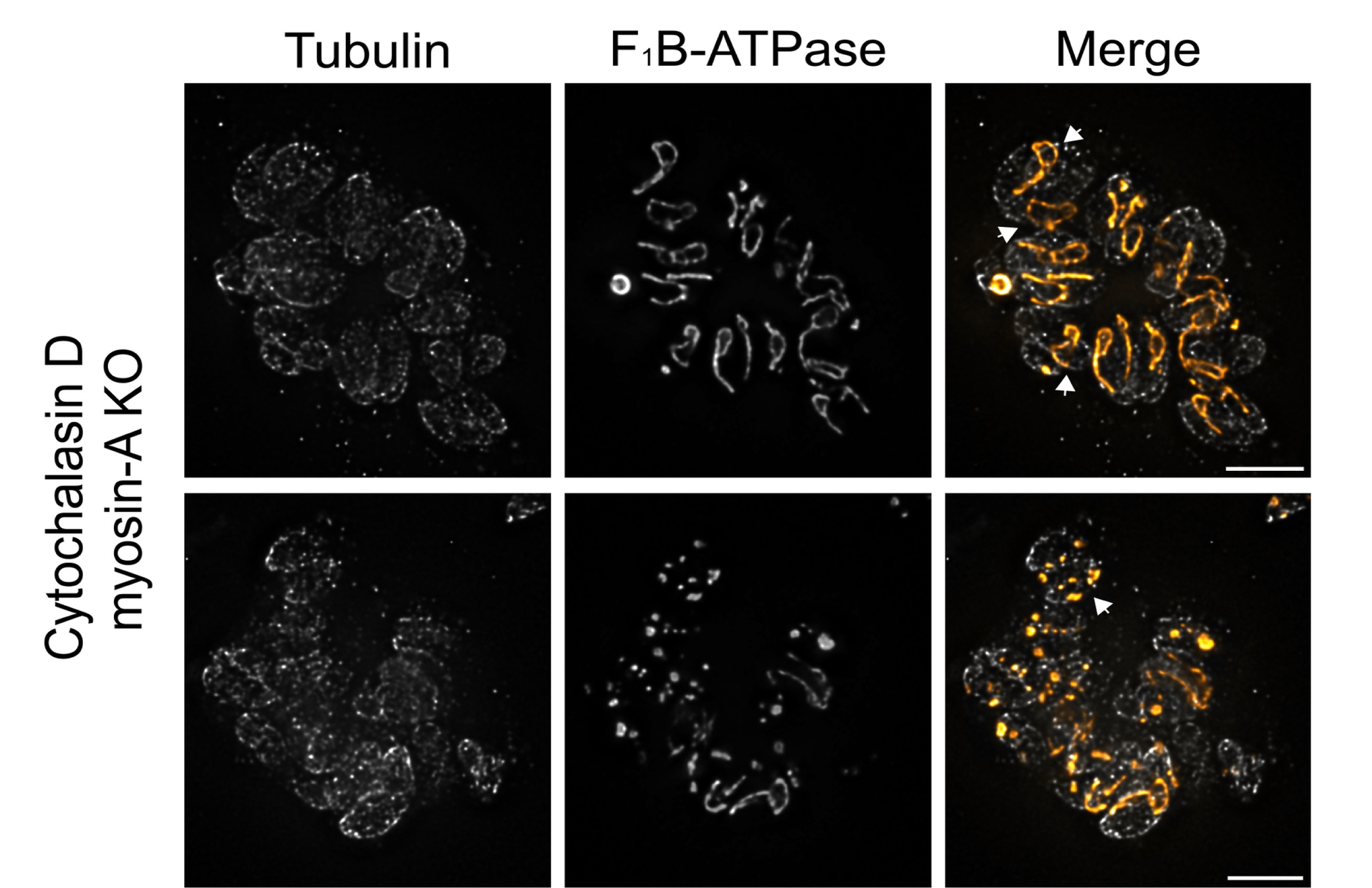

Supplement: S8 Fig — IFA of large parasite vacuoles grown in the presence of 1 mM of Cytochalasin D (A). Parasites were stained for Tubulin (gray) and F1B-ATPase (orange) Scale bar: 5μm. (TIF) [file ppat.1012127.s015.tif]

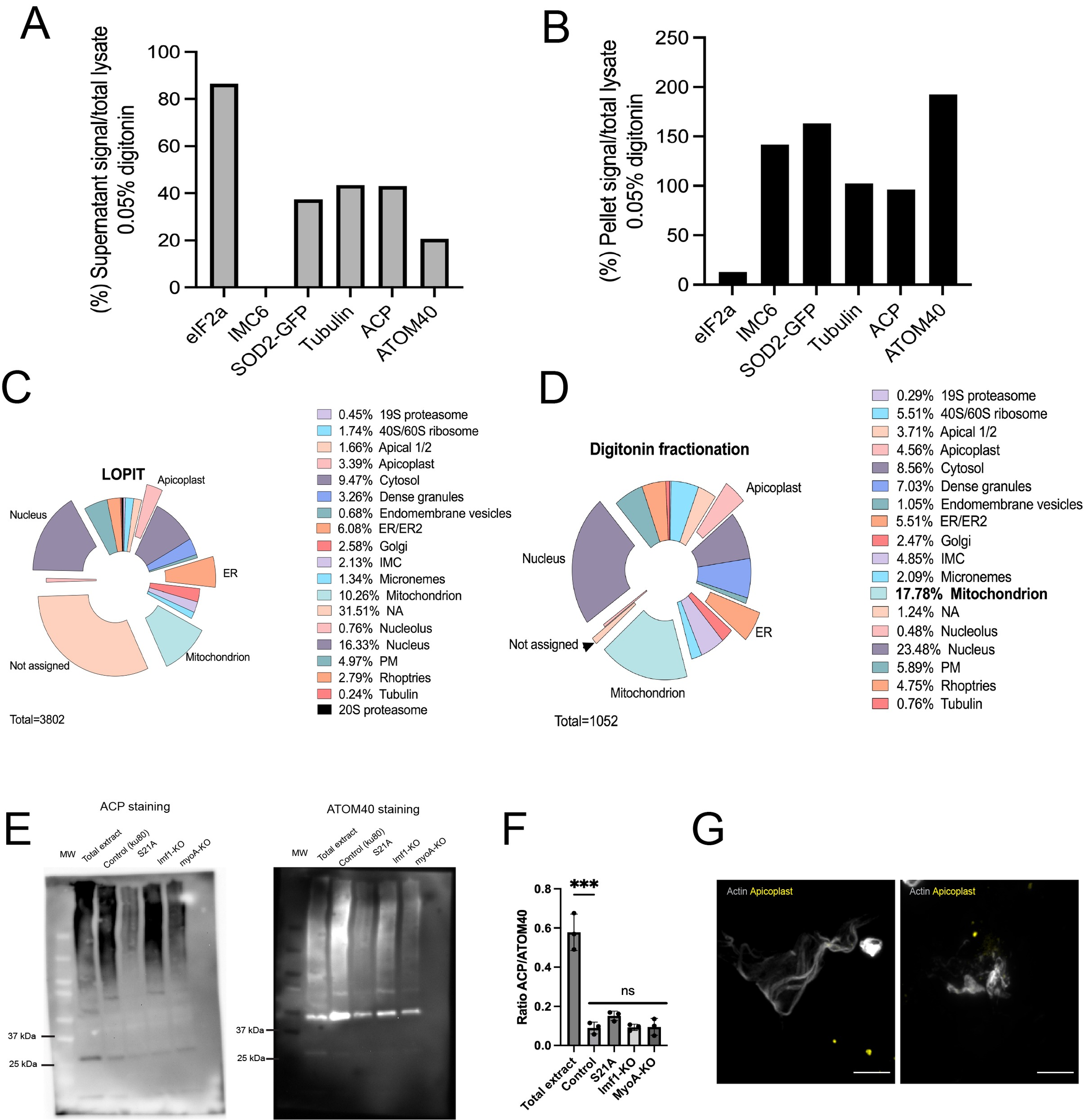

Supplement: S9 Fig — A. Densitometry of supernatants after treatment with 0.05% digitonin. B. Densitometry of pellets after treatment with 0.05% digitonin. C. Pie chart of hyperLOPIT data, showing the total distribution of proteins across cellular compartments. D. Pie chart of proteins identified by mass spectrometry from the digitonin fraction. E. Membranes of post-cushion enriched mitochondrion vesicles were probed for the presence of apicoplast (anti-ACP) and anti-ATOM40 (mitochondrion). F. Quantification of the ratio ACP7ATOM40 staining in each fraction by densitometry. Bar graphs represent the mean ±sd of each calculated ratio. G. Mitochondrion-enriched fractions isolated from all the cell lines were stained with anti-ACP (yellow), incubated with Phalloidin-488 labeled actin filaments (gray), and observed by fluorescence microscopy. (TIF) [file ppat.1012127.s016.tif]
